# Activation of nuclear factor kappa B in peripheral blood mononuclear cells from malaria patients

**DOI:** 10.1186/1475-2875-11-191

**Published:** 2012-06-10

**Authors:** Chuchard Punsawad, Srivicha Krudsood, Yaowapa Maneerat, Urai Chaisri, Noppadon Tangpukdee, Emsri Pongponratn, Kwannan Nantavisai, Rachanee Udomsangpetch, Parnpen Viriyavejakul

**Affiliations:** 1 Department of Tropical Pathology, Faculty of Tropical Medicine, Mahidol University, 420/6 Rajvithi Road, Bangkok, 10400, Thailand; 2 Department of Tropical Hygiene, Faculty of Tropical Medicine, Mahidol University, 420/6 Rajvithi Road, Bangkok, 10400, Thailand; 3 Department of Clinical Tropical Medicine, Faculty of Tropical Medicine, Mahidol University, 420/6 Rajvithi Road, Bangkok, 10400, Thailand; 4 Department of Microbiology, Faculty of Medicine, Srinakarinwiroj University, 114 Sukumvit 23, Bangkok, 10110, Thailand; 5 Department of Pathobiology, Faculty of Science, Mahidol University, 272 Rama VI Road, Ratchathewi, Bangkok, 10400, Thailand; 6 Center for Emerging and Neglected Infectious Diseases, Mahidol University, Bangkok, 10400, Thailand

**Keywords:** Malaria, *Plasmodium falciparum*, *Plasmodium vivax*, Nuclear factor kappa B, Peripheral blood mononuclear cells, Interleukin-10, Tumor necrosis factor

## Abstract

**Background:**

Malaria parasites and their products can activate a specific immune response by stimulating cytokine production in the host’s immune cells. Transcription nuclear factor kappa B (NF-κB) is an important regulator for the control of many pro-inflammatory genes, such as interleukin-1 (IL-1) and tumor necrosis factor (TNF). The activation and expression of NF-κB p65 in peripheral blood mononuclear cells (PBMCs) of malaria patients were investigated and correlated with the levels of IL-10 and TNF to study the nature of NF-κB p65 and its linkage to inflammatory cytokines.

**Methods:**

The sample group comprised 33 patients admitted with malaria caused by *Plasmodium vivax* (n = 11), uncomplicated *Plasmodium falciparum* (n = 11), and complicated *Plasmodium falciparum* (n = 11). Peripheral blood was collected at admission and on day 7 for PBMC isolation. Healthy subjects were used as a control group. The expressions of NF-κB p65 in the PBMCs from malaria patients and the plasma levels of IL-10 and TNF were measured by using enzyme-linked immunosorbent assay (ELISA). The immunofluorescence technique was used to determine NF-κB nuclear translocation.

**Results:**

At admission, patients with *P. vivax* and uncomplicated *P. falciparum* had significantly elevated phospho-NF-κB p65 levels in the PBMCs compared with those of healthy controls. However, patients with complicated *P. falciparum* malaria had decreased levels of phospho-NF-κB p65. On day 7 post-treatment, significantly increased phospho-NF-κB p65 was found in the PBMCs of patients with complicated *P. falciparum*, compared with healthy controls. The plasma level of IL-10 was elevated in day 0 in patients with complicated *P. falciparum* malaria and was found to be negatively correlated with phospho-NF-κB p65 level (*r*_*s*_ = −0.630, *p* = 0.038). However, there was no correlation between phospho-NF-κB p65 expression and TNF level in patients with complicated *P. falciparum* malaria.

**Conclusions:**

This is the first report demonstrating alterations in NF-κB p65 activity in the PBMCs of malaria patients. The altered lower features of NF-κB p65 in the PBMCs of patients with complicated *P. falciparum* at admission could be due to a suppressive effect of high IL-10 associated with complicated *P. falciparum* malaria.

## Background

Nuclear factor kappa B (NF-κB) plays a crucial role in immune and inflammatory responses, through the regulation of various genes involved in pro-inflammatory cytokines, adhesion molecules, chemokines, inducible enzymes, and apoptosis [1]. Mammals express five NF-κB protein members: NF-κB-1 (p50), NF-κB-2 (p52), Rel A (p65), Rel B, and c-Rel [2]. These proteins have a structurally conserved amino-terminal (300 amino-acid) region, containing dimerization, nuclear localization and DNA binding domains. In unstimulated cells, NF-κB is bound to its inhibitor, the inhibitor kappa B (IκB) protein, and it appears in the cytoplasm as an inactive form. Following stimulation, IκB is first phosphorylated by IκB kinase (IKK) and then rapidly degraded by the proteosome. Subsequently, activated NF-κB translocates into the nucleus, where it binds to the DNA regulatory site to regulate specific gene expressions [2,3].

The process of malaria pathogenesis is very complex and still poorly understood. Several studies have indicated that the expression of adhesion molecules on vascular endothelial cells and the production of pro-inflammatory cytokines are closely linked to the activation of NF-κB protein transcription. It is already known that circulating mononuclear cells are involved in the immune responses and production of pro-inflammatory cytokines. Accumulated evidence has demonstrated that excessive production of pro-inflammatory mediators leads to systemic and organ-related pathological conditions. Previous studies have reported that levels of endogenous pyrogens such as interleukin-6 (IL-6), IL-1β and IL-8 were elevated in *P. vivax* and *P. falciparum* malaria infections [4-7]. The increasing serum TNF levels were reported to be associated with increased mortality in Malawian children with severe malaria [8]. Plasma TNF levels were also found to be higher in Gambian children [9]. A recent study of experimental *P. falciparum* infection in malaria-naïve individuals has shown a coordinated increase in the level of pro-inflammatory cytokines, including IFN-γ, IL-12 and IL-8 in the serum [10]. Previous *in vitro* studies have demonstrated that haemozoin (HZ) [11] and glycosylphosphatidylinositol (GPI) [12,13] can stimulate monocytes and macrophages to synthesize pro-inflammatory cytokines via NF-κB pathways [12], leading to rapid phosphorylation of IκB, with subsequent nuclear translocation of NF-κB [13]. Using a microarray methodology, it has been reported that the transcripts of Toll-like receptor signaling through NF-κB pathways was significantly up-regulated in the peripheral blood mononuclear cells (PBMCs) of both experimentally and naturally acquired malaria infections [14]. Aside from the process of cytoadhesion and sequestration, the pathogenesis of malaria is associated with increased production of pro-inflammatory cytokines. Circulating mononuclear cells are known to be induced in the immune response, and are potent biological sensors of infection. No study has reported the expression of NF-κB in PBMCs from malaria patients and its association with circulating cytokines such as IL-10 and TNF. To elucidate this process, NF-κB p65 activity in the PBMCs of malaria patients was determined and correlated with the plasma levels of IL-10 and TNF as well as with pertinent clinical data.

## Methods

### Malaria patients and healthy controls

Thirty-three malaria patients admitted to the Hospital for Tropical Diseases, Faculty of Tropical Medicine, Mahidol University, Thailand, were included in this study. The patients were divided into three groups: (1) *Plasmodium vivax* malaria (n = 11), classified based on a positive blood smear with *P. vivax* by microscopic examination, (2) uncomplicated *Plasmodium falciparum* malaria (n = 11), classified based on a positive blood smear with *P. falciparum,* and no evidence of severe or complicated malaria, and (3) complicated *Plasmodium falciparum* malaria (n = 11), defined by the WHO criteria [15]. Complicated malaria was defined as patients exhibiting one or more of the following manifestations: hyperparasitaemia (> 250,000 parasite/μl), hypoglycaemia (glucose < 22 nmol/l), severe anaemia (haematocrit < 20% or haemoglobin < 7.0 g/dl), or increased serum level of creatinine of more than 3.0 mg/dl. Cerebral malaria was defined as unrousable coma with positive asexual forms of *P. falciparum* in blood smears, with other causes of coma excluded. Eleven healthy volunteers living in Bangkok, a non-endemic malaria area, were recruited as the control group. This group had no history of malaria infection. Written informed consent was obtained from all patients or their legal representatives before enrollment in the study. The study protocol was approved by the Ethics Committee, Faculty of Tropical Medicine, Mahidol University (MUTM 2010-053-01).

### Blood collection

Five milliliters (ml) of peripheral blood was collected in heparinized tubes from the malaria patients on day 0 (pre-treatment) and day 7 (post-treatment) for PBMC isolation. In addition, 20 ml of whole blood was obtained from the healthy controls for PBMC isolation, which were employed as unstimulated cells, to investigate the ability of malaria-patient sera to induce NF-κB activation. To prepare the sera from malaria patients, clotted blood was centrifuged at 1,700 g for 10 min. The supernatant representing the serum was harvested and stored in an aliquoted state at −80°C. The serum was heat-inactivated at 56°C for 30 min before use.

### Preparation of PBMCs

PBMCs were isolated from freshly heparinized blood by gradient centrifugation, using Isoprep® separation medium (Robbins Scientific, CA, USA), according to the manufacturer’s instructions. The heparinized blood samples were centrifuged at 1,700 g for 5 min and plasma was removed and stored at −80°C until use for cytokine measurements. The remaining blood samples were diluted with an equal volume of phosphate-buffered saline (PBS) (pH 7.4), layered carefully into a conical tube containing Isoprep® solution, then centrifuged at 1,200 g for 25 min. After centrifugation, the PBMCs were removed and washed three times with PBS. The cells were stained with trypan blue and counted with a haemocytometer to determine the number of viable PBMCs.

### Total protein extraction

PBMCs were suspended in ice-cold lysis buffer (Cell Signaling, MA, USA) (20 mM Tris–HCl, 150 mM NaCl, 1 mM Na_2_EDTA, 1 mM EGTA, 1% Triton, 2.5 mM sodium pyrophosphate, 1 mM β-glycerophosphate, 1 mM Na_3_VO_4_, 1 μg/ml leupeptin) and a protease inhibitor cocktail (Sigma-Aldrich, MO, USA) for 5 min. The lysates were briefly sonicated on ice, centrifuged at 14,000 g for 10 min at 4°C, and the supernatants were harvested. Protein concentrations in each sample were determined by Bradford assay (Pierce Biotechnology, IL, USA), using bovine serum albumin (BSA) as the standard.

### Measurement of total- and phospho-NF-κB p65

The expression of NF-κB p65 in the PBMCs was assessed by sandwich ELISA kit (Cell Signaling, MA, USA) according to the manufacturer's protocol. Total cell proteins (10 μg/well) were added into a 96-well microplate coated with total-/phospho-NF-κB p65 mouse monoclonal antibody and incubated for 2 h at 37°C. The plate was then washed with PBS containing 0.05% (w/v) Tween-20 (PBS-T) and 100 μl of total-/phospho-NF-κB p65 rabbit monoclonal antibody was added to the wells and incubated for 1 h at 37°C to detect the captured total-/phospho-NF-κB p65 protein. After washing with PBS-T, 100 μl of anti-rabbit IgG secondary antibody conjugated with horseradish peroxidase (HRP) was added and incubated for 30 min at 37°C. To develop the reaction, 100 μl of 3,3’,5,5’-tetramethylbenzidine (TMB) substrate (Cell Signaling, MA, USA) was added and incubated for 10 min at 37°C. Finally, 100 μl of 0.18 M sulfuric acid was added to stop the reaction. The optical density (OD) of the yellow-colored product was determined with a microplate reader at 450 nm. All assays were carried out in duplicate.

### Determination of NF-κB p65 nuclear translocation

The translocation of NF-κB p65 from the cytoplasm to the nucleus was examined by immunofluorescence. PBMCs were smeared on adhesive slides coated with 3-aminopropyltriethoxysilane (Sigma-Aldrich, MO, USA) and fixed with 3.7% formaldehyde in PBS (pH 7.4) for 20 min at room temperature. After fixation, the cells were permeabilized with 0.5% Triton X-100 for 10 min. After washing with PBS, the slides were blocked with 5% BSA for 30 min at room temperature and incubated with mouse anti-human NF-κB p65 (F-6) monoclonal antibody (1:200) (Santa Cruz Biotechnology, Santa Cruz, CA) for 1 h at 37 °C. The slides were then incubated with goat anti-mouse antibodies conjugated with Alexa-488 (1:1000) (Invitrogen, Carlsbad, CA) for 45 min at 37°C. Finally, the slides were mounted with FluorSave^TM^ reagent (Calbiochem, Nottingham, UK) and observed under a fluorescence microscope (Olympus BX 41, Tokyo, Japan) connected to a digital camera (Olympus DP 20, Tokyo Japan) and a standard UV filter set. Normal PBMCs stimulated with 50 ng/ml of TNF for 30 min were used as a positive control and the omission of primary antibody was used as a negative control. For quantitative analysis, PBMCs (approximately 200–300 cells) were randomly examined by fluorescence microscopy under high power (x400) and cells stained positive for nuclear NF-κB p65 were counted. To calculate the percentage of cells with NF-κB nuclear translocation, the number of positive nuclear stained cells was divided by the total number of cell and the result was multiplied by 100.

### Measurement of IL-10 and TNF in plasma of malaria patients

The levels of IL-10 and TNF in plasma was determined by the Human IL-10 and TNF ELISA Development Kit (Peprotech, NJ, USA), a quantitative sandwich enzyme immunoassay using a purified rabbit antibody against IL-10 or by TNF pre-coated onto an ELISA plate (Nunc Maxisorp F96, Roskilde, Denmark), and then incubated overnight at room temperature. Human recombinant IL-10 and TNF protein at serial concentrations and 2-fold diluted plasma samples were incubated into the wells. Assay standards and samples were added to duplicate wells in the plate, which was incubated for 2 h at room temperature. After washing, either a biotinylated purified rabbit anti-human IL-10 or TNF antibody as the detection antibody was added to each well at a concentration of 50 ng/ml and incubated for 2 h at room temperature. Then, an avidin-horseradish peroxidase (HRP) conjugate was added to the wells and incubated for 30 min at room temperature. For color development, 2,2'-azino-bis (3-ethylbenzothiazoline-6-sulfonic acid), or ABTS liquid substrate (Sigma-Aldrich, MO, USA), was added and incubated at room temperature. Absorbance was read at 405 nm with wavelength correction set at 650 nm. The sensitivity of the kit was within the range of 32–2000 pg/ml for IL-10 and 16–2000 pg/ml for TNF.

### Induction of NF-κB activation in healthy volunteer PBMCs by malaria patient sera

To investigate whether the sera from malaria patients could induce the expression of NF-κB p65 in PBMCs of healthy volunteers, PBMCs (2 x 10^6^ cells) were separately stimulated by 10% sera from healthy controls and patients with *P. vivax*, uncomplicated *P. falciparum,* and complicated *P. falciparum* malaria for 0, 30, and 60 min, at 37°C, 5% CO_2_. TNF (50 ng/ml) was used as positive control. After incubation, PBMCs were washed with PBS and total proteins were isolated to detect the phospho-NF-κB p65 using sandwich ELISA.

### Statistical analysis

Data were expressed as mean ± standard error of the mean (SEM). The normality of distribution was determined by the Kolmogorov-Smirnov test. Differences in NF-κB p65 levels between groups were compared by unpaired Student’s *t*-test. Student’s paired *t*-test was used to assess differences in NF-κB p65 within groups. Differences in IL-10 and TNF cytokine levels between groups were analysed by the Mann–Whitney test and the Wilcoxon signed-rank test was used to compare the difference in cytokine levels within groups between day 0 and day 7. In addition, the correlations within groups between levels of NF-κB p65 and pertinent clinical data including age, days of fever, malaria parasite density, RBC, WBC, haemoglobin, haematocrit, platelet, and cytokine levels were calculated using Spearman’s rank correlation (*r*_*s*_). Data was analysed by statistical analysis performed using SPSS version 17.0 software (SPSS, IL, USA). The *p* value < 0.05 was considered significantly different.

## Results

### Study subjects

The clinical and laboratory parameters of malaria patients and healthy controls are shown in Table 1. On admission, the mean parasite density was significantly higher in patients with complicated *P. falciparum* malaria compared to those with uncomplicated *P. falciparum* infection (*p* = 0.001). Patients diagnosed with *P. vivax* malaria were administered chloroquine and primaquine, whereas patients with *P. falciparum* malaria were treated with artesunate and mefloquine. On day 7 post-treatment, no asexual forms of malaria parasites were found in the peripheral blood of all malaria patient groups. Complications in patients with complicated *P. falciparum* malaria included pulmonary oedema (four of 11, 36.4%), cerebral malaria (six of 11, 54.5%), acute renal failure (six of 11, 54.5%), shock (three of 11, 27.3%), anaemia (six of 11, 54.5%), and acidosis (one of 11, 9.1%).

**Table 1:** Clinical and laboratory parameters of malaria patients and healthy controls

	***P. vivax***		**Uncomplicated *P. falciparum***		**Complicated *P falciparum***		**Healthy controls**
	**Day 0**	**Day 7**	**Day 0**	**Day 7**	**Day 0**	**Day 7**	
Age (years)	25 (18–36)		31 (18–55)		35 (18–57)		26 (21–33)
Sex (Male/Female)	11/0		10/1		10/1		7/4
Duration of fever (days)	3 (1–5)		5 (2–9)		6 (3–9)		None
Parasitaemia (/μl)	62,916	0	30,853	0	825,475^#^	0	None
WBC (10^3^/μl)	6.3	7.4	6.5	5.6	8.5^*^	8.5^*^	6.6
RBC (10^6^/μl)	4.9	5.0	4.1^*^	4.1	4.2^*^	3.6^*^	5.1
Haematocrit (%)	39.2^*^	39.3^*^	32.2^*^	32.0	34.0^*^	28.2^*^	44.6
Haemoglobin (g/dl)	13.2^*^	13.1	10.6^*^	10.4	11.6^*^	9.6^*^	14.3
Platelet (10^3^/μl)	97^*^	303	106^*^	353	36^*^	226^*^	245

Data presented as mean. (n = 11 for each group).

^#^Significant difference (*p* < 0.05) vs uncomplicated *P. falciparum* malaria.

^*^Significant difference (*p* < 0.05) vs healthy controls.

### Activation of NF-κB p65 in the PBMCs of malaria patients

**Figure 1:**
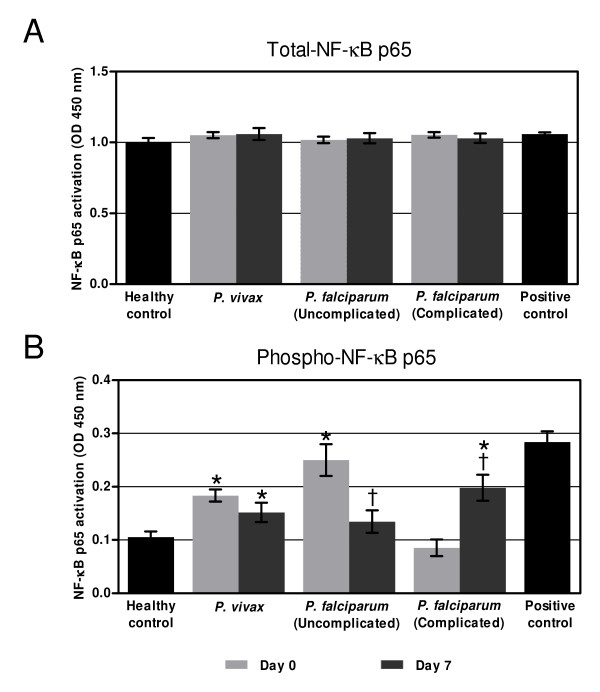
**Activation of NF-κB p65 in the PBMCs of malaria patients determined by ELISA.** (**A**) Total NF-κB level in the PBMCs of malaria patients (n = 5 for each group). No difference in total-NF-κB p65 level was observed in PBMCs of all malaria patient groups, *p* > 0.05 compared with healthy controls (Student *t*-test). (**B**) Phospho-NF-κB p65 levels in the PBMCs of malaria patients on day 0 and day 7 (n = 11 for each group). Protein extract from PBMCs stimulated with 50 ng/ml of TNF for 30 min was used as a positive control. *Significance of *p* < 0.05 compared with healthy controls (Student *t*-test)*,*^†^Significance of *p* < 0.05 compared with the day of admission (Student *t*-test). Data are presented as a mean ± SEM,.

The total-NF-κB p65 levels detected in the PBMCs was similar in all experimental groups (*p* > 0.05) on both day 0 and day 7 (Figure 1A). At admission, phospho-NF-κB p65 levels were significantly elevated in the PBMCs of patients with *P. vivax* (0.182 ± 0.04, *p* < 0.001) and uncomplicated *P. falciparum* malaria (0.250 ± 0.10, *p* < 0.001), compared to the healthy controls (0.104 ± 0.04). However, the phospho-NF-κB p65 levels were significantly lower in the PBMCs of patients with complicated *P. falciparum* malaria on admission compared to day 7 post-treatment (0.086 ± 0.06, *p* = 0.01). Among the patient groups, phospho-NF-κB p65 activity was significantly higher in the PBMCs of patients with uncomplicated *P. falciparum* malaria, compared to patients with *P. vivax* (*p* = 0.01) or complicated *P. falciparum* malaria (*p* = 0.001) (Figure 1B). On day 7 post-treatment, phospho-NF-κB p65 activity was significantly increased in the PBMCs of patients with complicated *P. falciparum* malaria, compared to the healthy controls (0.197 ± 0.03 vs 0.103 ± 0.01, *p* = 0.002) (Figure 1B). Levels of phospho-NF-κB p65 remained significantly elevated in PBMCs of the patients with *P. vivax* (0.151 ± 0.01), whereas phospho-NF-κB p65 levels in PBMCs from patients with uncomplicated *P. falciparum* malaria showed an increasing trend, but this was not statistically significant (*p* = 0.210) when compared to healthy controls (0.103 ± 0.01). In addition, no difference in the level of phospho-NF-κB p65 was found between uncomplicated and complicated *P. falciparum* malaria on day 7 (*p* > 0.05). No correlation was observed in any group of patients between the level of phospho-NF-κB p65 and other clinical parameters such as age, days of fever, malaria parasite density, RBC, WBC, haemoglobin, haematocrit, and platelet levels.

### Nuclear translocation of NF-κB p65 in the PBMCs of malaria patients

**Figure 2:**
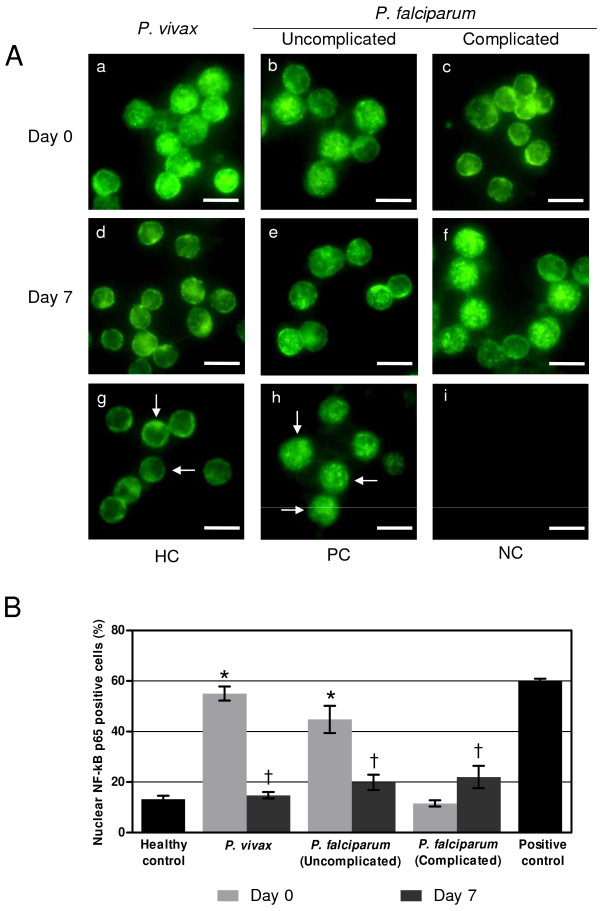
**Expression of NF-κB p65 in the PBMCs of malaria patients by immunofluorescence method.** (**A**) Representative immunofluorescence images of NF-κB p65 in the PBMCs of malaria patients on admission (a, b, c) and on day 7 post-treatment (d, e, f) compared with healthy controls (HC) as evidenced by cytoplasmic NF-κB p65 staining (arrows) (g) positive control (PC) showing nuclear NF-κB p65 staining (arrows) after stimulation with 50 ng/ml of TNF for 30 min (h) and negative control (NC) by omission of primary antibody (i). Bars = 10 μm. (**B**) Quantitative analysis of the NF-κB p65 activation in PBMCs of malaria patients. *Significance of *p* < 0.05 compared with healthy controls (Student *t*-test)*,*^†^Significance of *p* < 0.05 compared with the day of admission (Student *t*-test). Data are presented as a mean ± SEM.

The work performed in the study demonstrated and confirmed nuclear translocation of NF-κB in the PBMCs from malaria patients by immunofluorescence assay (Figure 2A). The percentages of NF-κB p65 nuclear staining with immunofluorescence were consistent with the findings of phospho-NF-κB p65 determined by ELISA. At admission, the mean percentage of cells with NF-κB p65 nuclear translocation was significantly increased in patients with *P. vivax* and uncomplicated *P. falciparum* malaria, compared to healthy controls (55.0 ± 2.8% vs 44.8 ± 5.4% vs 13.2 ± 1.3%, *p* < 0.001, *p* < 0.001, respectively) (Figure 2B). However, on day 7 the mean percentage of cells with NF-κB p65 nuclear translocation in all patient groups was similar to healthy controls (Figure 2B).

### IL-10 and TNF levels in the plasma of malaria patients

**Figure 3:**
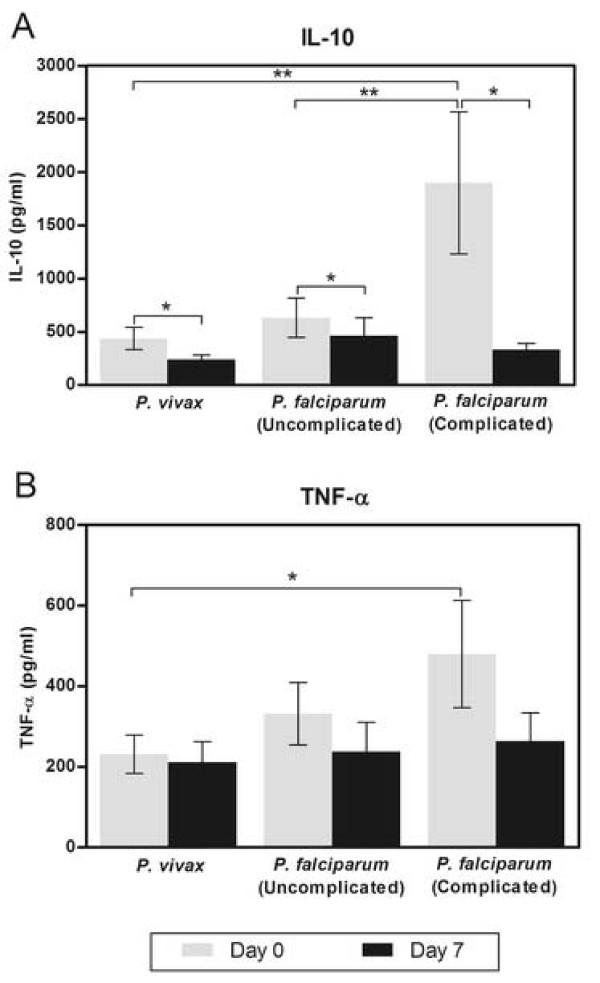
**Plasma concentrations of IL-10 and TNF in the patients with*P. vivax*, uncomplicated*P. falciparum*and complicated*P. falciparum*(n = 11 for each group) on day 0 and day 7.** (**A**) Plasma concentrations of IL-10 in three malaria patient groups. *Significance of *p* < 0.05 compared with the mean value determined on day 0, **Significance of *p* < 0.001 compared among different malaria groups. (**B**) Plasma concentrations of TNF in three malaria patient groups. *Significance of *p* < 0.05 compared among different groups. Data are presented as mean ± SEM.

The expression levels of IL-10 and TNF in the plasma of malaria patients are presented in Figure 3. Complicated *P. falciparum* malaria had strikingly higher plasma IL-10 concentrations than *P. vivax* (*p* < 0.001) and uncomplicated *P. falciparum* (*p* = 0.002) patients during the acute illness (day 0). The mean plasma IL-10 on day 0 for *P. vivax* was 437.44 ± 104.60 pg/ml as compared with uncomplicated *P. falciparum* (630.13 ± 183.78 pg/ml) and complicated *P. falciparum* malaria patients (1,898.80 ± 666.50 pg/ml) (Figure 3A). Nonetheless, levels of IL-10 in these malaria groups were significantly higher than those of the healthy controls (8.20 ± 5.96 pg/ml, all *p* < 0.001). On day 7, the mean plasma IL-10 in the three malaria groups declined significantly compared to their levels on day 0 (day 7; 238.43 ± 42.61 pg/ml for *P. vivax* (*p* = 0.003), 464.30 ± 169.00 pg/ml for uncomplicated *P. falciparum* (*p* = 0.041), and 331.26 ± 61.41 pg/ml for complicated *P. falciparum* malaria patients (*p* = 0.010)). The mean plasma levels of IL-10 in *P. vivax* patients were not significantly different from those observed in uncomplicated *P. falciparum* patients on both day 0 (*p* > 0.05) and day 7 (*p* > 0.05), and on day 7 of patients with complicated *P. falciparum* malaria (*p* > 0.05) (Figure 3A). Eventually, on day 7, circulating IL-10 levels were found to decline and returned to the same levels as for *P. vivax* and uncomplicated *P. falciparum*.

At admission, plasma TNF was markedly elevated in patients with *P. vivax* (231.08 ± 47.89 pg/ml), uncomplicated *P. falciparum* (331.70 ± 77.55 pg/ml), and complicated *P. falciparum* (479.64 ± 133.53 pg/ml), compared with the healthy controls (10.35 ± 10.02 pg/ml, all *p* < 0.001). The mean TNF concentration of patients with complicated *P. falciparum* was significantly higher than patients with *P. vivax* malaria (*p* < 0.05) (Figure 3B). TNF concentrations were monitored at day 7 and the levels were 211.19 ± 51.73 pg/ml, 237.65 ± 73.39 pg/ml and 263.95 ± 70.25 pg/ml for *P. vivax*, uncomplicated *P. falciparum* and complicated *P. falciparum* malaria patients, respectively. TNF concentrations did not differ within each group of malaria patients at admission and day 7 post-treatment (all *p* > 0.05) (Figure 3B). In addition, there was no correlation between the plasma levels of IL-10 and TNF in any malaria groups.

### Correlations between NF-κB p65 in PBMCs and plasma IL-10

In complicated *P. falciparum* malaria, a significant negative correlation between phospho-NF-κB p65 in PBMCs and plasma level of IL-10 (*r*_*s*_ = −0.630, *p* = 0.038) was established during acute illness. No correlation was observed between phospho-NF-κB p65 in PBMCs and plasma TNF in any of the malaria groups.

### Effect of malaria serum on NF-κB p65 in the PBMCs of healthy controls

**Figure 4:**
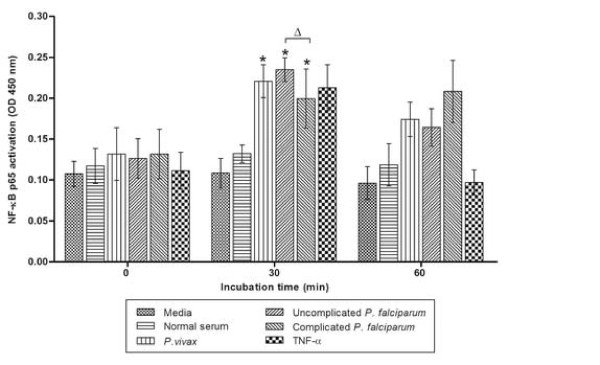
**Malaria sera-induced NF-κB p65 activation in the PBMCs of healthy controls (n = 5).** The protein extracts (10 μg/well) of the PBMCs from healthy controls after stimulation with serum from malaria patients were analysed for phospho-NF-κB level using ELISA. The results demonstrated significantly increased levels of phospho-NF-κB p65 in the PBMCs in response to malaria-patient sera after 30 min of stimulation with 10% serum from malaria patients with *P. vivax*, uncomplicated *P. falciparum,* and complicated *P. falciparum* malaria. TNF treatment was used as positive control and PBMCs treated with media was used as untreated or negative control. *Significance of *p <* 0.05 compared to normal serum treatments (Student *t*-test). ^Δ^Significance of *p <* 0.05 compared between groups. Data are presented as mean ± SEM.

The level of phospho-NF-κB p65 in the PBMCs increased significantly in response to sera from all malaria patient groups (*P. vivax*, uncomplicated *P. falciparum,* and complicated *P. falciparum* malaria) at 30 min after stimulation (Figure 4) with the highest stimulation by sera from uncomplicated *P. falciparum* malaria. In addition, the levels of phospho-NF-κB p65 activation induced by sera from patients with uncomplicated *P. falciparum* malaria were significantly higher than those induced by sera from patients with complicated *P. falciparum* malaria (*p* = 0.044). However, this effect seems to be transient. In the present study, the level of phospho-NF-κB p65 induced by sera from patients showed a tendency to return to the pre-stimulation state after 60 min (Figure 4).

## Discussion

This is the first report showing NF-κB expression in the PBMCs of malaria patients and its correlation with IL-10 and TNF by using sandwich ELISA. The use of ELISA has become a powerful method for measuring protein phosphorylation. ELISA is more quantitative than Western blotting and possesses high specificity and sensitivity due to the use of two antibodies specific for the target protein employed together in the sandwich. NF-κB p65 activation was increased in the PBMCs of *P. vivax* and uncomplicated *P. falciparum* patients, on both day 0 and day 7, whereas in complicated *P. falciparum* patients, elevated NF-κB p65 activity was observed only on day 7 post-treatment. NF-κB activation may be triggered by various ligands or proteins of malaria parasites that induce up-regulation of the NF-κB signaling pathway, leading to nuclear translocation of NF-κB and regulation of gene expression. It is possible that the increased NF-κB p65 levels in the PBMCs with malaria infection are involved in the enhancement of inflammatory cytokines. Consistent with the increased level of phospho-NF-κB p65 in the PBMCs, the immunofluorescence assay confirmed NF-κB p65 immunostaining in PBMC nuclei, indicating the active NF-κB protein state in malaria infection. Data from the literature of experimental *in vitro* malaria studies show that the mechanisms induced or involved in the activation of NF-κB p65 include haemozoin (HZ)-induced enhancement of inflammatory cytokines [11,16-18], activation of matrix metalloproteinase-9 (MMP-9) in human monocytes fed with trophozoites and HZ [19], and *P. falciparum* glycosylphosphatidylinositol (GPI) stimulating monocytes and macrophages, leading to the activation of NF-κB downstream signaling pathways induced expression of pro-inflammatory mediators, such as TNF, IL-6, IL-12, and nitric oxide (NO) [12,13]. Recent investigations studied the innate immune response in malaria infection, showing that Toll-like receptor 1 (TLR1), TLR2, and TLR4 were induced in PBMCs from both experimentally [14,20] and naturally acquired malaria infections [14]. These findings suggest that the activation of TLRs by GPI [21] and HZ [22] transmit signals in an intracellular pathway leads to the activation of transcription factor NF-κB, which in turn propagates a signal to the nucleus to regulate the expression of pro-inflammatory cytokines. Consequently, these actions could cause increased levels of phospho-NF-κB p65 and nuclear translocation of NF-κB p65 in the PBMCs of malaria patients.

NF-κB p65 activity was decreased in PBMCs from patients with complicated *P. falciparum* at admission, consistent with the reduced mean percentage of NF-κB p65 nuclear translocation evidenced by the immunofluorescence study. These findings agree with previous reports which demonstrated that PBMCs from patients with sepsis and major trauma reduced the active form of NF-κB p65 on the day of admission [23,24]. The silencing of NF-κB p65 gene expression reported in severe systemic inflammation may also explain the important signaling event in complicated *P. falciparum* wherein NF-κB p65 could be repressed by cytokines [25]. Studies have shown that immunosuppressives such as TGF-β [26,27] and IL-10 (also known as anti-inflammatory cytokine) [28] reportedly alter NF-κB expression and translocation, and contribute to cell desensitization [26-28]. Normally, IL-10 is produced by macrophages as well as T and B lymphocytes, and has been shown to play a significant role in immunoregulation, involving negative feedback on the production of pro-inflammatory cytokine [29]. It has been reported that increasing plasma IL-10 was detected in cerebral and severe malaria patients at admission, in contrast to the patients with uncomplicated *P. falciparum* malaria [30]. Elevated IL-10 levels have been detected in serum of Thai patients with acute *P. falciparum* malaria prior to treatment and the levels were found to return to normal after malaria treatment [31]. To investigate whether the decrease in nuclear translocation of NF-κB found in complicated *P. falciparum* malaria patients is linked to plasma IL-10 levels, IL-10 levels were determined in the malaria groups. In this study, the plasma level of IL-10 was significantly elevated in complicated *P. falciparum* malaria infection and had a negative correlation with phospho-NF-κB p65 expression at admission. When the IL-10 levels were high, the phospho-NF-κB p65 levels were low. This correlation was not found in *P. vivax* or uncomplicated *P. falciparum* infections. At admission, the plasma levels of IL-10 in patients with complicated *P. falciparum* malaria were 4.3 times and 3 times higher than in patients with *P. vivax* and uncomplicated *P. falciparum*, respectively. This observation suggests that decreased levels of NF-κB p65 in the PBMCs of complicated *P. falciparum* patients during acute infection could be due to a negative feedback loop mechanism, or the consequence of high levels of IL-10, an important anti-inflammatory cytokine associated with severe disease. It has been shown that IL-10 inhibits NF-κB activation rapidly and in a dose-dependent manner [28]. At day 7 post-treatment, the plasma levels of IL-10 in complicated *P. falciparum* malaria declined 5.7 times from the level on day 0, to the same levels as the *P. vivax* and uncomplicated *P. falciparum* malaria groups. This trend is similar to results in a previous report [30]. The lower level of IL-10 could explain the elevated level of activated NF-κB in the PBMCs from complicated *P. falciparum* malaria at day 7 post-treatment.

Furthermore, the study investigated whether malaria patient serum could induce NF-κB p65 activation in unstimulated PBMCs. Significantly, transiently increased levels of phospho-NF-κB p65 were found in the healthy PBMCs 30 min after stimulation with malaria serum, consistent with previous studies on endothelial cells [32], monocytes [33], and human cardiac myocytes [34]. However, the transient increased of phospho-NF-κB p65 in malaria sera-induced healthy PBMCS did not concur with the decline of NF-κB p65 at admission in complicated *P. falciparum* malaria. The PBMCs from healthy controls are naïve and activation is short-lived. The effect might not be long enough to initiate a complex response to malaria serum. In contrary, the PBMCs collected from complicated *P. falciparum* malaria patients was sensitized by prolonged infection and response mechanisms induced by other cell signaling processes. The present study elucidates that sera from malaria patients can induce NF-κB p65 activation in naïve PBMCs. It would be helpful to further analyse the expression of NF-κB in PBMCs from malaria patients after stimulation with malaria sera to determine the event of “desensitization” in malaria. Further work to investigate whether pre-incubation of the malaria sera with an anti-IL-10 neutralizing monoclonal antibody would suppress NF-κB activation in PBMCs from complicated *P. falciparum* patients will be of interest.

## Competing interests

The authors declare that they have no competing interests.

## Authors’ contributions

CP carried out the ELISA and immunofluorescence work, preliminary data analysis, and wrote the first draft of the manuscript. SK, NT and KN collected clinical data, supervised patient recruitment, critically analysed, and interpreted the data. YM, UC, EP and RU participated in the study design and manuscript preparation and revision. PV formulated the research idea, designed the experiments, gave laboratory and technical support, supervised, and revised the manuscript. All authors have approved the final version of the manuscript.
